# Glucagon-like Peptide-1 and Dual GIP/GLP-1 Receptor Agonists in Brain: Exploring the Expanding Role and Safety in Neuropsychiatry

**DOI:** 10.3390/ijms27083628

**Published:** 2026-04-18

**Authors:** Ana Cristina Tudosie, Loredana-Maria Marin, Simona Georgiana Popa, Andreea Loredana Golli

**Affiliations:** 1 Department of Endocrinology, University of Medicine and Pharmacy, 200349 Craiova, Romania; ana.dragomirescu@umfcv.ro; 2 Department of Pharmaceutical Physics, Faculty of Pharmacy, “Carol Davila” University of Medicine and Pharmacy, 020956 Bucharest, Romania; loredana-maria.marin@umfcd.ro; 3 Department of Diabetes, Nutrition and Metabolic Diseases, University of Medicine and Pharmacy, 200349 Craiova, Romania; 4 Department of Public Health and Healthcare Management, University of Medicine and Pharmacy, 200349 Craiova, Romania; andreea.golli@umfcv.ro

**Keywords:** GLP-1/GIP receptor agonists, neuroprotection, neuropsychiatry, safety, serotonin, dopamine

## Abstract

Glucagon-like peptide-1 (GLP-1) and dual GIP/GLP-1 receptor agonists, originally introduced for the management of type 2 diabetes mellitus and obesity, are increasingly recognized for their broader actions within the central nervous system, with emerging implications in neuropsychiatry and neurodegeneration. This review integrates current preclinical and clinical evidence, emphasizing their pharmacodynamic profile, central receptor distribution, and the molecular pathways linking metabolic signaling to neural function. Evidence suggests that GLP-1 receptor activation across key brain regions involved in energy balance and reward modulates multiple neurotransmitter systems, including dopamine and serotonin, as well as glutamatergic and GABAergic transmission, thereby influencing behavior, affective processes, and cognitive function. In parallel, these agents exhibit neuroprotective properties through improved neuronal insulin sensitivity, attenuation of neuroinflammatory pathways, and support of neuroplasticity, alongside effects on limiting pathological protein aggregation. Dual GIP/GLP-1 agonism may further potentiate these central actions through complementary metabolic and synaptic mechanisms. Although pharmacovigilance data have identified isolated neuropsychiatric adverse events, current clinical evidence does not support a consistent causal association. Collectively, incretin-based therapies represent a promising translational approach at the interface of metabolic and neuropsychiatric disorders, warranting further investigation into their long-term central safety, therapeutic efficacy, and clinical relevance.

## 1. Introduction

The gastrointestinal tract synthesizes two natural peptide hormones, glucose-dependent insulinotropic polypeptide (GIP) and glucagon-like peptide-1 (GLP-1), collectively referred to as incretins [1]. In recent decades, considerable scientific interest has focused on the enteroinsular axis and the physiological and pathophysiological mechanisms mediated by these hormones, owing to their crucial contribution to the regulation of insulin secretion and systemic glucose homeostasis [2]. Upon eating, these hormones are released into the bloodstream and attach to their target receptors [1]. GIP and GLP-1 are released from enteroendocrine K cells and L cells, respectively, following intestinal nutrient absorption of macronutrients such as glucose, amino acids and lipids [2].

GLP-1 is a 30- to 31-amino-acid peptide hormone derived from the tissue-specific post-translational processing of proglucagon, a 160-amino-acid precursor encoded by the GCG gene located on chromosome 2q24.2 [3]. In intestinal L-cells, predominantly located in the distal small intestine and colon, proglucagon is cleaved by prohormone convertase 1/3 (PC1/3) to generate GLP-1, along with other peptides such as GLP-2, oxyntomodulin, and peptide YY [3]. GLP-1 secretion exhibits a biphasic release pattern: an initial rapid rise within 15–30 min post-meal, mediated by neuroendocrine pathways (including vagal afferents and enteric neurotransmitters like acetylcholine), followed by a second minor peak at 90–120 min resulting from direct nutrient interaction with distal L cells [4,5,6,7].

Moving beyond systemic glucose control, GLP-1 and dual GIP/GLP-1 agonists are now recognized as central-acting neuropharmacological agents. This shift reflects their expanding use in obesity and T2DM, alongside growing evidence of their direct impact on the brain [8,9,10,11,12,13,14]. In addition to peripheral sources, GLP-1 is also synthesized in the central nervous system (CNS) by pre-proglucagon-expressing neurons in the nucleus of the solitary tract (NTS), which project to various brain regions involved in energy balance and reward [15,16,17].

The capacity of these agonists to influence neural circuits is mediated through their interaction with GLP-1 receptors (GLP-1Rs). GLP-1R belongs to the class B family of G protein-coupled receptors (GPCRs), signaling primarily through Gαs to increase cAMP levels within localized signalosomes [18]. These receptors are widely expressed across multiple regions implicated in energy homeostasis, reward processing, and autonomic control [19]. High densities of GLP-1Rs are found in the hypothalamic nuclei (arcuate—ARC, paraventricular—PVN, dorsomedial—DMH), critical for regulating appetite, as well as in the brainstem (NTS and area postrema) involved in satiety signaling [4,20]. Importantly, GLP-1Ra are also expressed in mesolimbic structures such as the ventral tegmental area (VTA), nucleus accumbens (NAc), amygdala, and hippocampus [15,21]. Within these circuits, GLP-1 signaling modulates dopaminergic activity and synaptic plasticity, effectively shifting the role of these agonists from simple metabolic regulators to potent neuropharmacological agents. By modulating dopaminergic activity and enhancing synaptic plasticity in these regions, GLP-1 signaling regulates reward-seeking behavior, reduces the salience of palatable foods, and influences emotional responses to stress, while simultaneously providing neuroprotective effects that support cognitive function [22,23,24,25,26].

The presence of GLP-1Rs in these reward-related brain regions suggests that GLP-1 signaling may play a significant role in modulating reward processing and addictive behaviors [27]. Mapping these precise neural interactions is essential for understanding how GLP-1Rs activation steers complex behaviors.

Beyond dopamine, these agonists influence other neurotransmitter systems. For instance, GLP-1Rs agonists modulate gamma-aminobutyric acid (GABA) signaling. Semaglutide has been shown to reduce alcohol consumption in rodents through alterations in central GABA neurotransmission [28,29]. Additionally, GLP-1Rs activation affects glutamatergic signaling, enhancing glutamate release in specific circuits like the cerebellar parallel fiber–Purkinje cell synapses via protein kinase A (PKA) signaling [30].

Although clinical observations show concurrent weight loss and neuroprotection, isolated reports of depressive symptoms and suicidal ideation have emerged [8,13,31,32,33,34,35,36], necessitating a clearer understanding of their central safety profile. Furthermore, while neuroprotective effects were strongly suggested by preclinical data, recent clinical evidence has been mixed. For instance, a recent Phase 3 trial [37] found that once-weekly exenatide did not significantly differ from placebo in providing disease-modifying benefits for Parkinson’s disease, suggesting that the translation of weight-loss-associated neuroprotection into definitive clinical outcomes remains a significant challenge.

This review aims to provide a comprehensive synthesis of the pleiotropic effects of GLP-1 and dual GIP/GLP-1 receptor agonists. Firstly, we examine the pharmacological characteristics and classification of these agents, highlighting the structural evolution from short-acting analogues to prolonged-release dual agonists like tirzepatide. Subsequently, we explore the neurotransmitter modulation induced by these drugs, focusing on their influence on dopamine, serotonin, glutamate, and GABA systems. We further analyze the distinct mechanisms of weight loss, differentiating between central satiety signaling and peripheral gastric effects. A key segment of this review is dedicated to the neuroprotective potential of these agonists in bridging the gap between type 2 diabetes mellitus (T2DM) and neurodegenerative disorders such as Alzheimer’s and Parkinson’s disease. Finally, we address the emerging neuropsychiatric risk profile, discussing the balance between metabolic benefits and mood-related adverse events.

## 2. Physiology of Incretin Hormones

GLP-1Rs are expressed in brainstem regions implicated in respiratory rhythmogenesis, including the nucleus tractus solitarius (NTS), locus coeruleus, and the pre-Bötzinger complex [38,39]. Their activation has been shown to stabilize breathing patterns and enhance respiratory drive in preclinical models, specifically by modulating chemosensory responses in the retrotrapezoid nucleus [40,41]. These effects may be particularly relevant in patients with comorbid obesity and obstructive sleep apnea, conditions frequently co-occurring with substance use disorders such as alcoholism and opioid dependence [42,43]. Experimental mapping using in situ hybridization has confirmed dense GLP-1R expression in medullary and pontine respiratory centers, where their stimulation improves pulmonary function and reduces mortality in obesity-induced respiratory pathophysiology [44,45]. Additionally, the bidirectional relationship between sleep disturbances and addiction has been increasingly recognized: substance use disrupts sleep architecture, while sleep disorders themselves may predispose to relapse and worsen addiction outcomes [46]. This interplay underscores a broader neuromodulatory role, suggesting GLP-1 receptors agonists (GLP-1RAs) as potential candidates for treating addiction-related sleep impairments.

While GIP and GLP-1 traditionally act as endocrine mediators for glucose-dependent insulin secretion in the pancreatic islets [47], their significance in neuropsychiatry arises from the integration of these peripheral signals within the central nervous system. The incretin effect [48], although progressively attenuated in metabolic disorders or increased body mass index [49,50,51], serves as a physiological template for how nutrient-related signals can modulate broader neural circuits, including those governing mood and behavior.

The increase in intracellular cAMP levels activates not only PKA but also Epac2 [18,52], which together phosphorylate CREB to drive gene transcription for β-cell survival [52]. The receptor’s efficacy is highly dependent on its spatial orientation. Ligands that favor plasma membrane signaling over internalization recruit less β-arrestin, resulting in sustained insulin release with reduced side effects [53]. While β-arrestin desensitizes the receptor by blocking G protein coupling, a process whose absence prolongs insulinotropic responses [54], it also acts as a scaffold to activate MAPK signaling (like ERK via c-Src [55]). This β-arrestin-mediated pathway, which is particularly critical for gene expression at pharmacological doses [56], promotes long-term cell survival and gene regulation independent of G protein signaling. In this context, transcriptomic studies (RNA-seq on rat islets and INS-1 832/3 cells) show that GLP-1 changes key DEGs. It increases IRS2, PDX-1, MAFA, and NKX6-1 expression, and elevates the anti-apoptotic factors BCL2 and BCL-XL. To protect cells against ER stress, GLP-1 signaling modulates the ATF4/CHOP pathway, typically leading to a decrease in the pro-apoptotic CHOP. Through β-arrestin-2, GLP-1 stimulates c-SRC, ERK1/2, and ELK1, leading to FOS and JUN expression [9]. Finally, reducing β-arrestin recruitment decreases receptor internalization and prolongs the expression of INS and anti-apoptotic genes [54,57].

Recent structural studies reveal that ligand-specific conformations of class B GPCRs modulate β-arrestin recruitment, receptor trafficking, and signaling duration, thereby influencing both therapeutic outcomes and side effect profiles [58]. This structural basis underlies ligand-specific signaling bias, rather than a simple binary selectivity.

Over the past years, substantial advances have been achieved in identifying the neural circuits involved in energy homeostasis. In particular, several hypothalamic nuclei, including the arcuate, paraventricular, ventromedial, and dorsomedial nuclei, as well as key brainstem structures such as the area postrema, nucleus tractus solitarius, and lateral parabrachial nucleus, have been recognized as critical components in the control of meal initiation and termination, thereby contributing to the regulation of energy balance and body weight maintenance [59,60].

GIP and/or GLP-1 receptors have also been identified in additional brain regions, where they appear to participate in several neural processes, including anti-apoptotic signaling, synaptic plasticity, and cognitive functions such as memory formation [61,62]. These receptors have been implicated in neural circuits associated with reward processing [61] and emotional regulation [61,63], supporting the hypothesis that incretin-based signaling may exert neuroprotective effects and potentially contribute to therapeutic strategies for various neurodegenerative disorders (vide infra).

These observations should be interpreted with caution due to interspecies differences. Specifically, variations in the distribution and expression patterns of GLP-1 receptors within the brain have been reported between rodents and higher-order species [61], as summarized in Table 1.

Unlike the stable receptors, the physiological hormone GLP-1 has an extremely short half-life because it is rapidly cleaved by dipeptidyl peptidase-4 (DPP-4) and neprilysin. This rapid degradation has driven the development of long-acting GLP-1RAs, which provide sustained receptor activation and improved therapeutic efficacy [4,48,69,70].

Within the central nervous system, GLP-1Rs are broadly distributed in neural circuits that integrate energy balance, autonomic regulation, and behavioral responses [4,49,50,70,71]. Their anatomical distribution aligns with the projection pathways of preproglucagon neurons originating in the NTS, which provide dense innervation to brain regions critical for metabolic and stress-related signaling [19,72].

Prominent receptor expression has been reported in hypothalamic nuclei that coordinate feeding behavior and energy homeostasis, including the arcuate, paraventricular, dorsomedial, and lateral hypothalamic areas [61,65,73]. In addition, GLP-1Rs are strongly expressed in brainstem structures such as the area postrema, NTS, and the dorsal motor nucleus of the vagus, where they participate in the modulation of auto-nomic and visceral functions [61,65,74].

Beyond hypothalamic and brainstem circuits, GLP-1 receptors are also detected in higher brain regions, including cortical areas, the hippocampus, caudate putamen, and globus pallidus, indicating potential involvement in cognitive processing and behavioral regulation [41,48]. In the spinal cord, GLP-1R expression is particularly enriched in sympathetic preganglionic neurons, where GLP-1 released from brainstem projections likely represents the primary endogenous ligand [65,72,74]. Despite the well-characterized distribution of these receptors, further investigation is required to clarify the specific functional roles of GLP-1 signaling across different neural networks.

## 3. Central Nervous System Distribution of GLP-1 Receptors

At the level of the NAc, receptor activation acts as a rheostat for dopaminergic signaling and may alter synaptic plasticity within reward circuits, contributing to reduced reward sensitivity and hedonic feeding. Some evidence suggests that GLP-1 signaling may also attenuate reward hypersensitization associated with addictive behaviors [75,76].

In the prefrontal cortex, GLP-1R activation appears to increase GABAergic tone and inhibitory postsynaptic signaling, particularly in the infralimbic cortex. Through these mechanisms, GLP-1 signaling may influence executive control and cognitive processes, with emerging evidence suggesting potential relevance for neurodegenerative disorders such as Alzheimer’s disease [28,68].

Central effects are sustained through direct neural projections from GLP-1-producing brainstem neurons. These neurons integrate peripheral metabolic cues via vagal afferents and project directly to the VTA and NAc, where GLP-1R activation decreases food intake and body weight, particularly suppressing consumption of highly palatable foods [77,78]. GLP-1R activation in CNS regions associated with motivation and reinforcement suggests a mechanistic bridge between gut-derived metabolic signals and neuropsychiatric regulation.

### 3.1. GLP-1R in Mesocorticolimbic Reward and Relapse Circuits

Within reward circuits, GLP-1 signaling appears to modulate mesolimbic dopaminergic activity in a context-dependent manner, attenuating reward-related dopaminergic hyperactivity while preserving baseline dopaminergic tone [24,76,79]. These effects are associated with alterations in phasic dopamine release and downstream adaptations within reward circuits, including changes in D1 receptor-expressing medium spiny neurons and synaptic plasticity mechanisms implicated in craving and relapse [76,79,80]. At the synaptic level, GLP-1R activation in the VTA modulates neuronal excitability through mechanisms involving glutamatergic and GABAergic signaling, including enhanced AMPA/kainate receptor-mediated transmission and regulation of local interneuron activity [15,24,28,75,81]. Similar mechanisms have been observed in the NAc, where GLP-1R signaling influences excitatory transmission in medium spiny neurons, suggesting that modulation of mesolimbic excitability may occur primarily through glutamatergic control rather than direct dopaminergic inhibition [82].

In these reward-related regions, GLP-1 signaling has been associated with increased inhibitory synaptic activity, indicating enhanced GABAergic tone in circuits that regulate affective processing and extinction learning [28]. Anatomically, GLP-1-producing neurons located in the NTS project directly to both the VTA and NAc, providing a functional pathway through which peripheral metabolic signals can influence central reward processing [15]. This integrated signaling pathway underscores how GLP-1 influences reward salience and the neurobiology of craving [76].

### 3.2. Gut–Brain Axis and Vagal Modulation of Craving

Communication between the gastrointestinal tract and the central nervous system is mediated by the gut–brain axis, a bidirectional signaling network involved in the regulation of energy homeostasis, reward processing, and motivational behavior. Within this system, GLP-1 functions as an important integrative signal. It is synthesized both in intestinal enteroendocrine L-cells and in preproglucagon neurons located in the NTS, allowing peripheral metabolic information to be conveyed to central circuits that regulate appetite and behavior [73,83].

Signals originating from the gut reach the brain predominantly through vagal afferent pathways. These fibers transmit sensory information from the gastrointestinal tract to the NTS, a key brainstem structure that also contains GLP-1 producing neurons. From there, glutamatergic projections extend toward components of the mesolimbic reward system, including the VTA and NAc, providing a functional link between peripheral metabolic signals and dopaminergic reward circuitry [84,85].

Evidence from experimental models indicates that GLP-1 receptor signaling within the NTS contributes to the regulation of reward-related behaviors, identifying this region as an important integration center for craving-related processes [84,85]. When vagal signaling is disrupted, these regulatory effects are lost, highlighting the essential role of intact gut–brain communication in this pathway [86]. Moreover, the effectiveness of this signaling system may vary between individuals. Differences in vagal tone, often assessed through measures such as heart rate variability, as well as metabolic factors including high-fat dietary patterns, can influence vagal responsiveness and thereby modify the impact of gut-derived signals on reward circuitry [87,88].

### 3.3. Synaptic Mechanisms

Activation of GLP-1Rs influences synaptic transmission within key structures of the mesocorticolimbic reward pathway. In the VTA, GLP-1 receptor stimulation enhances AMPA/kainate-type glutamatergic input to dopaminergic neurons, altering neuronal excitability and reducing the salience of conditioned re-ward-related cues [81]. In the NAc, GLP-1 receptor activation in-creases presynaptic glutamate release and attenuates cue-induced reinstatement of re-ward-seeking behavior, potentially through modulation of glutamatergic or GABAergic interneurons [76,81].

These mechanisms suggest that GLP-1 receptor signaling may exert anti-craving ef-fects by modifying synaptic plasticity within addiction-related neural circuits. Synaptic efficacy in the VTA and NAc plays a crucial role in drug-induced neuroadaptations, and modulation of GLP-1 receptors may help restore the excitatory–inhibitory balance involved in these processes [79,80].

## 4. Pharmacological Characteristics of GLP-1 and GLP-1/GIP Agonists

Beyond their endogenous counterparts, pharmacological GLP-1RAs are engineered to resist rapid enzymatic degradation, allowing for sustained systemic and central effects [13,89,90,91].

Upon binding to G-protein-coupled receptors, GLP-1 exerts effects beyond simple glucose homeostasis and satiety [92]. In the CNS, it plays a vital role in maintaining neuronal integrity by regulating synaptic plasticity, modulating neurotransmitter release, and attenuating neuroinflammation [93]. Consequently, the activation of central GLP-1 pathways is increasingly recognized for its potential to restore impaired neurogenesis and correct synaptic dysfunction, offering a mechanistic basis for its therapeutic investigation in depressive disorders and cognitive decline [13,92].

### 4.1. Classification and Clinical Profile

To date, several GLP-1RAs have received regulatory approval for the management of T2DM, cardiovascular disease, and obesity. These agents include albiglutide, dulaglutide, exenatide (both standard and extended-release formulations), liraglutide, lixisenatide, and semaglutide (Table 2). Distinct from the others, which require subcutaneous injections, semaglutide can be taken by mouth, typically as a daily tablet [8].

Clinically, GLP-1RAs offer substantial clinical benefits with a favorable cardiovascular safety profile, showing no increased risk of pancreatitis or pancreatic cancer [94]. However, it is worth noting that gastrointestinal adverse events remain common [95]. These agents are safe in chronic kidney disease, reduce severe hypoglycemia, and lower major cardiovascular risks in patients with established disease [90]. While limited outcome data restricts the broader use of oral semaglutide [96], GLP-1RAs demonstrate comparable efficacy and safety in post-transplant diabetes [97].

**Table 2:** Pharmacokinetic and Clinical Characteristics of GLP-1 and Dual GIP/GLP-1 Receptor Agonists.

Agonist Type	Agent	Trade Name	Elimination Half-Life	Approved Indication (s)	Route and Frequency of Administration	Sources
**GLP-1RAs**	Albiglutide	Tanzeum	5 days	T2DM	Subcutaneous injection, once-weekly (30 mg/50 mg)	[98,99]
	Dulaglutide	Trulicity	5 days	T2DM	Subcutaneous injection, once-weekly (0.75 mg/1.5 mg)	[99,100]
	Exenatide IR	Byetta	2.4 h	T2DM	Subcutaneous injection, twice-daily (5 μg/10 μg)	[101,102]
	Exenatide ER	Bydureon	2 weeks	T2DM	Subcutaneous injection, once-weekly (2 mg)	[103,104]
	Liraglutide	Victoza	13 h	T2DM	Subcutaneous injection, once-daily (0.6 mg/1.2 mg/1.8 mg)	[105,106]
	Lixisenatide	Lyxumia/Adlyxin	2.8 h	T2DM	Subcutaneous injection, once-daily (10 μg/20 μg)	[107,108]
	Semaglutide	Ozempic	145–168 h	T2DM	Subcutaneous injection, once-weekly (0.25 mg/0.5 mg/1 mg/2 mg)	[109,110,111,112]
		Wegovy	145–168 h	Chronic weight management/obesity	Subcutaneous injection, once-weekly (0.25 mg/0.5 mg/1 mg/1.7/2.4 mg)	
		Rybelsus	153–161 h	T2DM	Oral doses, once-daily (3 mg/7 mg/14 mg)	
**Dual GIP/GLP-1RAs**	Tirzepatide	Mounjaro	116.7 h	T2DM	Subcutaneous injection, once-weekly (2.5 mg/5 mg/7.5 mg/10 mg/12.5 mg/15 mg)	[113,114]

### 4.2. Dual GLP-1/GIP Agonists (Tirzepatide)

Expanding the therapeutic approach, the role of GIP is significant. It enhances nutrient-stimulated insulin secretion [90,115] and exerts a complex, glucose-dependent effect on glucagon, suppressing it in hyperglycemia while stimulating counterregulatory release during hypoglycemia [116]. This modulation is often diminished in T2DM [117]. However, pharmacological activation of GIP supports weight loss and improves insulin sensitivity by optimizing lipid storage in adipose tissue and reducing ectopic muscle fat [115,116]. Consequently, the complementary actions of GIP and GLP-1 provide a strong rationale for dual agonists. These agents aim to leverage synergistic effects for improved glycemic control and weight reduction [90].

As the only Food and Drug Administration -approved dual GIP/GLP-1 receptor agonist, tirzepatide offers potent co-agonism. Structurally, tirzepatide is a prototypical single-molecule dual agonist engineered from the native GIP sequence. Its conjugation to a C20 fatty acid extends its half-life to approximately 116 h, allowing weekly dosing without adjustments for renal or hepatic impairment [118,119,120].

Crucially, the optimization of its pharmacokinetic profile, marking a shift away from short-acting analogues, has been pivotal for enhancing both efficacy and tolerability. Beyond glycemic control, tirzepatide has been shown to significantly reduce liver fat content and improved apnea-hypopnea indices in obstructive sleep apnea [90]. Cardiovascular outcomes proved non-inferior to dulaglutide [121], while the SURMOUNT program confirmed its efficacy in obesity management following lifestyle interventions [122]. Their use is expanding from treating T2DM and obesity to mitigating cardiovascular disease events [1].

### 4.3. Central Integration

The therapeutic impact of these agonists on mood and behavior is predicated on their ability to cross the blood–brain barrier (BBB) [8,13,123]. This interaction effectively bridges metabolic and emotional regulation. At the cellular level, this activation triggers intracellular pathways (such as cAMP and PI3K) that modulate dopamine and serotonin transmission while regulating synaptic plasticity. Furthermore, GIP efficacy appears to rely on downstream GLP-1 receptor crosstalk, integrating these signals to centrally regulate appetite, reward, and cognition [124].

## 5. Neuromodulatory and Neurotransmitter Effects of GLP-1/GIP Agonists

GLP-1Rs can modulate depolarization-induced release of several neurotransmitters, including GABA, glutamate, serotonin, and dopamine, in the cortex and hippocampus (Table 3). This broad modulatory capacity serves as the basis for understanding how these agents influence both metabolic and neuropsychiatric outcomes [13].

However, the clinical outcome of this modulation is region-specific and dose-dependent. In brain regions involved in emotional regulation, such as the amygdala, hypothalamus, and insula, excessive or prolonged receptor activation has been associated with neuropsychiatric adverse effects, including worsening depression, anxiety, and apathy [125,126].

### 5.1. The Dopaminergic System

Recent evidence has demonstrated a close interaction between the nigrostriatal dopaminergic system and metabolic regulation in humans [127]. Specifically, dopamine release in mesolimbic reward circuits is attenuated by GLP-1 activation, reducing food-related reinforcement. Essentially, these agents discourage eating for pleasure by lowering the gratification obtained from food [8,128,129].

### 5.2. The Serotonergic System

Beyond dopaminergic modulation, these agents enhance serotonergic tone, a key mediator of emotional processing in the amygdala and hippocampus. Specifically, GLP-1Rs expressed on serotonergic neurons directly increase neurotransmitter turnover, thereby alleviating anxiety-like behaviors [8,13].

Furthermore, central serotonin availability can be indirectly assessed via the tryptophan-to-kynurenine ratio. In obesity-driven inflammation, the enzyme IDO-1 is upregulated, shunting tryptophan away from serotonin synthesis and toward the kynurenine pathway. By suppressing systemic cytokines, GLP-1 and GIP agonists may inhibit IDO-1 activity, thereby restoring the tryptophan-to-kynurenine balance and enhancing endogenous serotonin production [130].

Neuroimaging studies support the presence of disturbances in both basal and stimulus-induced neurotransmitter activity in obesity, where reduced postprandial serotonergic activity may impair satiety signaling [131,132,133,134].

### 5.3. GABA and Glutamate

Beyond monoamines, GLP-1RA may enhance cognition by restoring disrupted glutamatergic and GABAergic neurotransmission, which are often implicated in conditions like schizophrenia. For instance, cognitive deficits have been linked to N-Methyl-D-Aspartate receptor hypofunction, reduced GluN1 expression, impaired glutamate uptake, and altered inhibitory signaling. Preclinical studies show that GLP-1RAs can normalize glutamate uptake, increase N-Methyl-D-Aspartate receptor subunit expression, and enhance GABA signaling in key brain regions, likely through downstream pathways shared with insulin receptor signaling [36].

Given that clinical depression is characterized by a marked reduction in GABAergic activity, the ability of these agonists to restore the excitatory–inhibitory balance is of significant therapeutic interest [13].

In this context, dual GLP-1/GIP agonists offer a distinct therapeutic advantage. Since GIP signaling also plays a critical role in modulating synaptic plasticity and preserving hippocampal integrity, the combined activation of both receptors may provide a synergistic restoration of the GABA-glutamate balance. This dual mechanism suggests that agents like tirzepatide could offer superior efficacy in normalizing the neurochemical deficits underlying mood disorders compared to GLP-1 monotherapy alone [13,135].

### 5.4. Brain-Derived Neurotrophic Factor and Neurogenesis

Neuroplasticity, driven primarily by brain-derived neurotrophic factor (BDNF), is essential for mitigating neuronal apoptosis. Since BDNF deficiencies are often precursors to depressive symptoms, restoring its levels remains a key therapeutic target [13,136]. In this context, exendin-4 has emerged as a potent compound capable of stimulating neurogenesis and neural proliferation [13,137,138].

However, dual GLP-1/GIP agonists may offer superior neurotrophic efficacy. Since GIPRs are also densely expressed in the hippocampus and are critical for synaptic plasticity, the simultaneous activation of both pathways is hypothesized to induce a synergistic upregulation of BDNF. Consequently, dual agonists like tirzepatide could provide more robust protection against neuronal atrophy and depressive symptoms than GLP-1 mono-therapy alone [135].

**Table 3:** Central effects of GLP-1/GIP receptor agonists on key neurotransmitter systems.

Neurotransmitter System	Central Effects Mediated by GLP-1/GIP RA	Functional Outcome	Sources
Dopaminergic System	Modulation of dopamine signaling in mesolimbic reward circuits	Reduction in food-related reinforcement and lowering gratification from food	[8,127,128,129]
Serotonergic System	Enhancement of serotonergic tone	Regulation of mood and potential improvement in depressive behaviors	[8,13]
Glutamate & GABA	Modulation of excitatory (glutamatergic) and inhibitory (GABAergic) neurotransmission	Restoration of synaptic plasticity; Potential cognitive enhancement (memory/learning)	[13,36]
Neurotrophic Factors	Upregulation of BDNF expression	Promotion of neurogenesis, neural proliferation, protection against neuronal atrophy and depressive symptoms	[135,136,137]

The widespread distribution of GLP-1 receptors in these regions provides the anatomical substrate for their diverse effects in neuropsychiatry. By targeting the hippocampus and prefrontal cortex, these agents can modulate cognitive functions and emotional resilience, which are frequently impaired in depressive and neurodegenerative disorders.

## 6. Nutrient-Induced Changes in Plasma Dopamine and Their Relationship with GLP-1 Signaling

Although plasma dopamine functions primarily as a peripheral regulator, its study is indirectly related to the main focus of this review as it reflects the systemic dopaminergic tone that interacts with central reward circuits. Recent evidence suggests that peripheral dopamine may act as an anti-incretin signal, creating a feedback loop that could influence central appetite control and emotional regulation, both of which are core components of neuropsychiatric health [139,140,141,142].

Dopamine has recently emerged as a key peripheral regulator of intestinal motility, renal sodium handling, and pancreatic β-cell function [143,144,145,146,147,148]. Specifically, it may act as an anti-incretin signal by activating D2 receptors, which in turn inhibits insulin secretion stimulated by both glucose and GLP-1 [143,149,150].

Peripheral dopamine originates from multiple sources, including the adrenal glands, sympathetic nerve terminals, and the gastrointestinal tract, particularly the pancreas, highlighting its integration within nutrient-responsive pathways [143,149,150]. Notably, plasma dopamine concentrations increase following mixed-meal ingestion in both humans and rodents, reflecting its sensitivity to nutritional stimuli [143,151].

This postprandial increase is predominantly driven by carbohydrate intake, with glucose inducing rapid and dose-dependent elevations in circulating dopamine levels, whereas lipids and proteins exert comparatively minor effects. These observations suggest the involvement of tightly regulated gut-derived nutrient-sensing mechanisms. This temporal rise in plasma dopamine aligns with incretin secretion, suggesting a functional synchrony between dopaminergic and GLP-1 signaling. In peripheral tissues, dopamine further modulates glucose metabolism independently of insulin signaling in skeletal muscle (via D1 receptors) and liver (via D2 receptors), while enhancing insulin-mediated glucose uptake in white adipose tissue through D2 receptors-dependent mechanisms [25]. However, this coordinated postprandial dopaminergic response is altered in metabolic disease, as dopamine excursions are attenuated in diabetic states despite the persistence of peripheral dopaminergic signaling components, suggesting a functional decoupling in metabolic disease.

## 7. Weight-Loss Mechanisms of GLP-1 and Dual GIP/GLP-1 Agonists

### 7.1. Central and Gastrointestinal Mechanisms of Satiety

GIP-R agonism appears to influence body weight through complex and context-dependent pathways. While GIP can stimulate lipogenesis in adipocytes, central GIPRs activation has been shown, in preclinical models, to increase c-Fos activity in hypothalamic feeding circuits, leading to reduced food intake in rodents [1]. Simultaneously, GLP-1-RAs lower body weight by acting both centrally and peripherally. In the hypothalamic satiety center, GLP-1 stimulates anorexigenic POMC/CART neurons and suppresses orexigenic neuropeptide Y and agouti-related peptide pathways, producing a strong feeling of fullness that curtails food intake [1]. Consequently, the combined dampening of dopaminergic reward and hypothalamic appetite centers produces enhanced satiety and decreased caloric intake [8,129,152].

Regarding the peripheral gastrointestinal effects, GLP-1 slows digestion by reducing gastric motility and increasing pyloric tone. This process relies on the vagus nerve sending sensory signals from the gut to the brain [1,153,154]. Interestingly, the body is much more sensitive to GLP-1’s effect on the stomach than its effect on hunger. It takes a substantially higher dose (up to 10,000×) to trigger satiety than to slow gastric emptying [155]. While GLP-1 acts locally on these nerves before being broken down, GIP does not appear to engage this specific vagal-mediated pathway for slowing gastric emptying [1,153,154].

Through this mechanism, peripheral GLP-1 also slows gastric emptying, blunting post-prandial glucose spikes and extending the period of gastric distention, which further reduces appetite [156,157,158]. Only short-acting GLP-1RAs provide a sustained reduction in gastric emptying [159]. Long-acting agents (including tirzepatide [158]) trigger tachyphylaxis, defined as a progressive attenuation of effect caused by continuous 24 h receptor exposure [158,159,160,161]. Comparative studies verify that while both classes reduce body weight, short-acting agents, like lixisenatide, are superior to long-acting ones (like liraglutide) in maintaining delayed gastric emptying and controlling post-meal glucose excursions [159].

### 7.2. Metabolic Synergy in Adipose and Hepatic Tissues

Beyond gastric modulation, chronic GLP-1 exposure impacts systemic energy expenditure by raising sympathetic outflow via the area postrema and rostral ventrolateral medulla, increasing lipolysis and fatty-acid oxidation in adipose tissue [1], and elevates circulating adiponectin, a hormone that improves insulin sensitivity and contributes to reductions in fat mass [162].

In contrast, GIP acts directly on fat cells to promote efficient storage (anabolic effects). It facilitates the clearance of circulating lipids and their safe storage in adipose tissue, thereby improving insulin sensitivity and preventing fat buildup in organs [154,163]. GLP-1, by comparison, acts indirectly through central mechanisms, signaling the nervous system to promote fat oxidation (catabolic effects) by increasing sympathetic tone and fatty-acid utilization [1]. The combination of GIP-mediated lipid storage capacity and GLP-1-induced lipid oxidation contributes to the maintenance of metabolically healthy adipose tissue. This synergy significantly boosts adiponectin levels, with tirzepatide demonstrating a marked 16–23% increase in a clinical trial conducted by Lee et al. [162]. Additionally, GIP-mediated enhancement of adipose-tissue insulin sensitivity promotes efficient triglyceride clearance and, when combined with GLP-1 signaling, amplifies overall energy-balance regulation [164,165].

These metabolic benefits extend to the liver. Despite the absence of GIP and GLP-1 receptors on hepatocytes, these incretins exert profound indirect effects on hepatic metabolism, largely mediated by adiponectin signaling [166,167]. Secreted by adipose tissue, adiponectin activates the hepatic cAMP/pAMPK pathway, mimicking a fasting-like metabolic state [168]. This signaling cascade upregulates beta-oxidation, via carnitine palmitoyl transferase-1 while suppressing de novo lipogenesis, through downregulation of SREBP-1c and fatty acid synthase [168]. Consequently, hepatic glucose production and steatosis are reduced, resulting in improved insulin sensitivity [166,167,168]. Furthermore, GLP-1 contributes by delaying gastric emptying, which limits the postprandial spike in intestinal lipid absorption. Pharmacodynamically, while GLP-1 monotherapies (such as dulaglutide) reduce atherogenic markers (apoC-III, apoB), tirzepatide further distinguishes itself by dose-dependently increasing lipoprotein lipase activityan, an effect likely attributable to its GIP component [163,169,170].

### 7.3. From Mechanisms to Clinical Outcomes

These mechanistic differences translate into a clear clinical hierarchy of weight loss efficacy. From least to most effective, the agents are ranked as follows: albiglutide < lixisenatide < exenatide < dulaglutide < liraglutide < semaglutide < tirzepatide [1]. Structural pharmacology explains some of these disparities: large molecules like albiglutide and dulaglutide have limited capacity to cross the blood–brain barrier, resulting in weaker stimulation of the brain’s satiety centers [169,170,171]. Specifically, tirzepatide demonstrates the most potent effect, with placebo-corrected weight loss reaching up to 11.6 kg in a T2DM trial conducted by Jastreboff et al. [169]. These findings indicate that dual GIP/GLP-1 receptor agonists, such as tirzepatide, exploit additive central appetite suppression and complementary peripheral lipid-handling mechanisms, resulting in greater weight loss than GLP-1-only therapies, as demonstrated in the phase 3 trial conducted by Frías et al. [172].

## 8. Neuroprotective Actions of GLP-1 and GIP Agonists in Neurodegeneration

Understanding intracellular signaling pathways like cAMP and PI3K/Akt is essential for evaluating GLP-1RA efficacy in neuropsychiatry, as these pathways directly drive neuronal survival and synaptic plasticity. In the context of severe mental illness, standard antipsychotic therapies are often limited by significant metabolic adverse effects, including increased risks of T2DM and cardiovascular disease [8]. Traditional medications generally fail to ameliorate the cognitive impairments that frequently accompany such disorders [31,34,35,123,173]. To address these metabolic and cognitive challenges, research has turned to incretin-based therapies (Table 4).

GLP-1 agonists improve cerebral insulin signaling, mitochondrial function and suppress neuroinflammation, thereby protecting dopaminergic neurons in Parkinson’s disease and reducing β-amyloid accumulation in Alzheimer’s disease [8,31,174,175,176]. Specifically, semaglutide’s neuroprotection is thought to arise from several inter-related actions on brain GLP-1Rs and downstream pathways [35]. Activation of central GLP-1Rs enhances neuronal insulin signaling and reduces insulin resistance, which supports cell survival, synaptic transmission, and attenuates apoptotic pathways [177].

By contrast, a deficiency in these receptors is associated with accelerated neurodegenerative processes, whereas their enhanced expression fosters neuroprotection and improved cognition. These pleiotropic properties suggest that GLP-1RAs may represent promising candidates to slow the progression of aging-related pathologies, particularly Alzheimer’s and Parkinson’s disease [8].

### 8.1. The Pathophysiological Interplay Between Type 2 Diabetes and Parkinson’s Disease

GLP-1 and GIP are incretin hormones that can cross the blood–brain barrier and activate their respective G-protein-coupled receptors on neurons and glial cells [178,179]. In the MPTP mouse model of Parkinson’s disease, dual incretin signaling restores impaired brain insulin sensitivity, a key contributor to neurodegeneration. In addition, GLP-1/GIP agonism dampens chronic neuroinflammation: astrocytic Glial Fibrillary Acidic Protein (GFAP) and microglial Iba-1 activation are reduced to near-control levels, indicating attenuated gliosis. This anti-inflammatory effect, together with decreased oxidative stress, helps preserve neuronal integrity. Functionally, treated mice show markedly improved motor coordination on the rotarod and greater grip strength compared with both MPTP-only and liraglutide-treated groups [178].

Further supporting this disease-modifying potential, preclinical mouse models of Parkinson’s disease, GLP-1 agonism attenuated motor deficits and protected dopamine neurons, suggesting a disease-modifying potential [180]. The same class reaches measurable brain concentrations, and activation of GLP-1 receptors is thought to counteract insulin resistance and α-synuclein aggregation that contribute to neurodegeneration [179,181]. For instance, in Parkinson’s models, semaglutide lowers α-synuclein aggregation, boosts expression of glial-cell-line-derived neurotrophic factor (GDNF) and increases tyrosine-hydroxylase activity, thereby preserving dopaminergic neurons and restoring dopamine levels [182].

However, dual agonists may confer superior neuroprotective efficacy. In mouse models of Parkinson’s disease, the dual GLP-1/GIP agonists DA-JC1, DA-JC4 and DA-CH5 rescued motor activity, protected dopaminergic neurons, increased dopamine synthesis, and reduced chronic microglial/astrocyte inflammation more effectively than the single GLP-1 analogue liraglutide [183]. They also lowered pro-apoptotic BAX/Bcl-2 ratios, activated the autophagy marker Beclin-1, and decreased oxidative stress, providing a multimodal neuroprotective profile that exceeds that of GLP-1 monotherapy [184,185].

Moving to clinical evidence in humans, results have been heterogeneous but encouraging. The phase-2 LIXIPARK trial, conducted by Meissner et al. [179], enrolled 156 participants with early Parkinson’s disease and gave daily sub-cutaneous lixisenatide (10 µg to 20 µg) for 12 months. At 12 months the on-medication MDS-UPDRS-III score improved by −0.04 points in the lixisenatide arm versus a 3.04-point worsening with placebo, a between-group difference of 3.08 points (95% CI 0.86–5.30; *p* = 0.007). After a 2-month washout, off-medication scores remained lower (17.7 vs. 20.6) in the active group, indicating a modest slowing of motor disability progression. The primary benefit appears linked to neuroprotective signaling rather than symptomatic dopamine replacement, but gastrointestinal toxicity (nausea 46%, vomiting 13%) limited tolerability and led to dose reductions in many participants. Larger and longer-duration trials are required to confirm efficacy and to explore whether dual GLP-1/GIP agonists might amplify these mechanisms.

Thus, GLP-1/GIP agonists act in Parkinson’s disease by restoring insulin signaling, enhancing neurotrophic support, suppressing neuroinflammation, and preserving dopaminergic neuronal integrity and motor function.

### 8.2. The Pathophysiological Interplay Between Type 2 Diabetes and Alzheimer’s Disease

T2DM markedly increases the risk of Alzheimer’s disease, with epidemiologic data indicating a 50–60% higher incidence in diabetic patients [175]. This strong correlation is rooted in the fact that insulin in the CNS regulates multiple molecular and physiological processes, including neuronal glucose transport, calcium signaling, tau phosphorylation, and amyloid-β clearance, as well as higher-order functions such as learning and memory. The concept of CNS insulin resistance arose from evidence of impaired insulin action in the brain, which may result from reduced insulin availability or decreased responsiveness at the level of insulin receptors [8,176,186,187,188,189,190].

This insulin resistance is not confined to peripheral tissues. It also occurs in the brain and is strongly associated with the progression of Alzheimer’s disease. In Alzheimer’s disease patients, the brain shows reduced insulin-receptor (IR) density, impaired IR signaling, and lower insulin levels in the cerebrospinal fluid, even when peripheral glucose homeostasis is normal [176]. The central mechanism driving this pathology is chronic insulin resistance, which impairs neuronal glucose uptake and creates a state of functional hypoglycemia, triggering oxidative stress and mitochondrial dysfunction [175].

This metabolic dysfunction is exacerbated by transport deficits at the level of the BBB. Insulin crosses the barrier via a saturable transporter. However, conditions such as aging, inflammation, or altered triglyceride levels can diminish this transport, lower brain insulin availability, and exacerbating IR in Alzheimer’s disease brains [191,192,193,194]. This deficit has immediate proteomic consequences, as reduced insulin signaling also diminishes the activity of insulin-degrading enzyme, a key protease that clears amyloid-β. When insulin-degrading enzyme is competitively inhibited by excess peripheral insulin, amyloid-β accumulates and forms pathological plaques [175].

To address the metabolic collapse described above, recent research has focused on GLP-1 and GIP RAs [195,196,197,198]. Originally developed for peripheral glycemic control in T2DM, these incretin mimetics have demonstrated a profound ability to cross the blood–brain barrier and restore insulin signaling pathways within the CNS [195]. A critical advantage of newer dual GLP-1/GIP agonists is their faster BBB penetration compared with liraglutide, leading to superior protection of dopaminergic and hippocampal synapses. As demonstrated in MPTP, 6-OHDA, and APP/PS1 mouse models, this enhanced penetration facilitates restoration of striatal dopamine, rescuing long-term potentiation, lowering amyloid-β and tau phosphorylation, and improvement of memory and motor performance [195].

These preclinical findings align with results observed in humans, where exendin-4 and liraglutide slowed gray-matter loss and cognitive decline in Alzheimer’s disease. Notably, these benefits persisted after drug washout, confirming disease-modifying effects [195].

Beyond chronic neurodegenerative conditions, dual GLP-1/GIP agonists like tirzepatide have also demonstrated strong protective effects against acute ischemic stroke. Unlike single-agent GLP-1 analogues, which often exhibit low brain-to-plasma ratios, the fatty-acid modification and balanced dual receptor activation of tirzepatide appear to facilitate moderate BBB penetration. Recent preclinical evidence has shown that tirzepatide significantly mitigates stroke-induced BBB disruption, cuts infarct volume, and restores neurological scores. At the molecular level, these benefits are driven by the restoration of the tight-junction protein Claudin-1 and the upregulation of C/EBP-α expression in both cortical tissue and human brain microvascular endothelial cells. Consequently, by preserving BBB integrity through C/EBP-α-mediated Claudin-1 transcription, dual agonists like tirzepatide represent promising adjunct therapies for acute ischemic injury [199].

The therapeutic relevance of GLP-1RAs, such as liraglutide, lies in their ability to attenuate T2DM-associated brain insulin resistance. In the study by Gejl et al. [196], liraglutide prevented the decline in cerebral glucose metabolism observed in the placebo group, indicating preserved neuronal glucose utilization even in patients with longer disease duration.

Moreover, GLP-1 restores insulin signaling in the brain, counteracting amyloid-β–induced insulin desensitization and supporting key enzymatic steps of neuronal glucose metabolism. As cerebral glucose hypometabolism is closely linked to cognitive decline and neurodegeneration, these findings suggest that GLP-1 receptor agonists target a central metabolic mechanism in Alzheimer’s disease, although cognitive benefits remain inconclusive in this pilot study [196].

Ultimately, GLP-1/GIP agonists counteract diabetes-induced brain insulin resistance by delivering growth-factor-like signals across the BBB, restoring synaptic plasticity and metabolic homeostasis, and mitigating the molecular hallmarks that link T2DM to Alzheimer’s disease.

Beyond insulin signaling alone, the accelerated production of advanced glycation end products (AGEs) via the “Maillard reaction” represents a further mechanistic link between T2DM and Alzheimer’s disease [175,200]. While AGEs accumulate during normal aging, their formation is significantly exacerbated by diabetic hyperglycemia, leading to the pathological modification of amyloid-beta plaques and neurofibrillary tangles. Consequently, monitoring toxic AGE levels in the CNS may provide a critical biomarker for the early detection of Alzheimer’s disease in diabetic populations [200,201].

GLP-1 and GIP RAs counteract this process by enhancing incretin signaling [202] and reducing oxidative stress, thereby suppressing the hyperglycemia-driven AGE accumulation. Specifically, these agents inhibit the pro-inflammatory AGE- Receptor for AGE signaling axis by activating cAMP pathways and downregulating Receptor for AGE expression [203]. Furthermore, by improving systemic glycemic control, these agonists reduce the substrate for the Maillard reaction and decrease the transport of peripheral AGEs into the CNS. The potential upregulation of soluble Receptor for AGE as a decoy receptor further limits the toxic modification of amyloid-β plaques and neurofibrillary tangles. While not direct Alzheimer’s disease treatments, GLP-1/GIP agonists mitigate a critical metabolic risk factor for neurodegeneration, reinforcing the role of CNS AGE levels as a valuable early biomarkers in diabetic populations [203,204].

In addition to AGEs, amyloid-beta-derived diffusible ligands (ADDLs) act as potent neurotoxins that induce brain-specific insulin resistance by disrupting synaptic signaling. Due to their high diffusivity, ADDLs bind to synapses and alter their conformation, significantly reducing the binding affinity between insulin and its receptors. This disruption impairs signal transduction and triggers a cascade of pathological events, including oxidative stress, synaptic loss, and tau hyperphosphorylation. Given their role in compromising insulin signaling, a phenomenon often termed “brain diabetes”, ADDL levels represent a promising diagnostic biomarker for Alzheimer’s disease [205,206].

Furthermore, a primary pathogenic parallel exists between the two conditions regarding the systemic deposition of amyloid-beta and pancreatic islet amyloid, both of which disrupt glucose homeostasis and beta-cells function [207,208]. The abnormal cleavage of APP generates amyloid-beta plaques, which activate GSK-3 and JNK signaling pathways [175]. While GSK-3 drives tau hyperphosphorylation and neurofibrillary tangle formation in both diseases, JNK activation, regulated by Islet Brain 1, promotes oxidative stress and beta-cells apoptosis. These shared molecular drivers, particularly GSK-3 and JNK, represent critical therapeutic targets linking metabolic and neurological degeneration [207,209].

This degenerative cycle is further fueled by systemic inflammation, characterized by elevated IL-6, C-reactive protein, and alpha1-antichymotrypsin. These mediators overlap with the inflammatory profile of Alzheimer’s disease, where C-reactive protein is a known risk factor. Addressing this link, PPAR-gamma agonists have been shown to reduce Alzheimer’s disease incidence by suppressing these cytokines, demonstrating that targeting shared inflammatory pathways can simultaneously mitigate insulin resistance and neurodegeneration [175,210].

In line with these findings, modern pharmacological agents like semaglutide further support neuroprotection. In Alzheimer’s models, semaglutide reduces amyloid-β accumulation by enhancing autophagy and inhibiting neuronal apoptosis [211]. Moreover, it attenuates oxidative stress, inflammation and programmed cell death in MPTP-treated mice, further supporting neuronal survival [212].

The convergence of these pathways suggests that modulating insulin and inflammatory signaling represents a unified frontier for treating neurodegeneration.

GLP-1RAs and GIP-GLP-1 receptor co-agonists represent promising therapeutic avenues for neurodegenerative diseases like Alzheimer’s disease and Parkinson’s disease by targeting the shared pathological processes [174,213].

Dual GLP-1/GIP agonists activate both the GLP-1R and GIPR in the brain, providing a broader growth-factor stimulus than GLP-1 alone. This combined signaling re-sensitises insulin pathways, lowers hyper-phosphorylated IRS-1/2 and restores PI3K-Akt signaling, which is essential for neuronal survival and glucose utilization [198,214,215]. Simultaneous GLP-1 and GIP receptor activation amplifies downstream cAMP/PKA/CREB and PI3K-Akt cascades, leading to higher expression of neurotrophic factors such as BDNF and GDNF, and stronger activation of pAkt/CREB signaling than liraglutide alone [198,216,217].

Collectively, these mechanisms, enhanced insulin signaling, dopaminergic support, anti-inflammatory/anti-oxidant effects, and promotion of autophagy, underlie the increasingly recognized neuroprotective benefits of metabolic therapies in Parkinson’s and Alzheimer’s disease. Dual GLP-1/GIP agonists consistently demonstrate superior synaptic preservation, reduced amyloid and tau pathology, and improved cognitive and motor outcomes in preclinical models.

**Table 4:** Mechanisms and Therapeutic Benefits of GLP-1/GIP Agonists in Neuroprotection.

Pathological Target	Mechanism of Action Induced by GLP-1/GIP Agonists	Therapeutic Benefit	Sources
Insulin Signaling	Activation of PI3K-Akt pathway; Restoration of insulin receptor sensitivity	Restores neuronal glucose transport; Improves cell survival and synaptic transmission	[176,213,214,215]
Neuroinflammation	Reduction in astrocytic (GFAP) and microglial (Iba-1) activation	Decreases chronic gliosis and release of pro-inflammatory cytokines (IL-6, TNF-α)	[177,182]
Protein Aggregation	Promotion of autophagy (Beclin-1 activation); Reduction in GSK-3β activity	Clearance of amyloid-β plaques (Alzheimer’s disease) and α-synuclein aggregates (Parkinson’s disease)	[181,210,218]
Oxidative Stress	Inhibition of JNK-mediated pathways; Improvement of mitochondrial function	Prevention of neuronal apoptosis and protection of dopaminergic neurons	[183,184,211]

## 9. Assessing the Neuropsychiatric Risk of GLP-1/GIP RA

Given the inherent link between metabolic disorders and psychiatric comorbidities, the neuropsychiatric safety of GLP-1RAs has become a focal point of recent investigation. Large-scale randomized controlled trials, including the SCALE [219] and STEP [220] programs, consistently report that depression and suicidal ideation are rare (<1%), with incidences comparable to placebo [219,220,221,222,223,224].

Beyond controlled clinical settings, a large-scale real-world cohort study published by Wang et al. [221] which analyzed over 240,000 patients, found that semaglutide was associated with a significantly lower risk of suicidal ideation compared to non-GLP-1 anti-obesity medications, contradicting concerns of increased psychiatric risk. These findings are further supported by pooled evidence, as recent meta-analyses [223] synthesizing data from multiple randomized controlled trials have consistently shown that the incidence of psychiatric adverse events in GLP-1RA treated groups does not differ statistically from control groups. Similarly, a comprehensive cohort study conducted by Shapiro et al. [222] comparing GLP-1RA users against active comparators (SGLT-2 and DPP-4 inhibitors) found no evidence of increased suicidality, reinforcing the favorable safety profile observed in both randomized controlled trials and real-world studies. Furthermore, following extensive reviews of pharmacovigilance data, both the Food and Drug Administration [225] and European Medicines Agency [226] concluded that current evidence does not support a causal link, suggesting that isolated reports may be confounded by baseline psychiatric comorbidities rather than the drug’s mechanism of action.

However, a discrepancy exists between these controlled findings and real-world spontaneous reporting. As clinical trials are often constrained by stringent eligibility criteria and limited duration, real-world pharmacovigilance data may offer broader insights. In this context, post-marketing reports have suggested potential signals of mood alterations.

Post-marketing pharmacovigilance has indeed produced safety signals for mood-related disorders among users of GLP-1 RA. Analysis of the Food and Drug Administration Event Reporting System from 2004 Q1 to 2023 Q1 identified 8240 psychiatric adverse-events reports, representing 4.55% of all GLP-1 RA-related adverse-events. While semaglutide showed a marginally higher proportion of reported psychiatric adverse events (5.82%) relative to other GLP-1 receptor agonists, tirzepatide exhibited the lowest proportion (2.71%). This difference may reflect its more recent approval and the limited duration of post-marketing surveillance. The most frequently reported psychiatric adverse-events were insomnia, anxiety, nervousness, depression (770 reports) and stress. Although early clinical trials did not show a clear link between GLP-1RAs therapy and psychiatric outcomes, the European Medicines Agency’s thyroid-cancer alert prompted an investigation of a possible suicide risk, which has not been confirmed by large trial data. Disproportionality analysis revealed eight psychiatric adverse-events categories with significantly elevated reporting odds ratios (e.g., nervousness ROR 1.97, binge eating ROR 2.70, self-induced vomiting ROR 3.77) indicating a statistical association with GLP-1 RA exposure [227].

This trend of mood destabilization is further corroborated by specific analyses of suicidal behavior in Europe. Following emerging case reports, the European Medicines Agency began a dedicated safety review of the whole class after reports of suicidal thoughts in patients using liraglutide and semaglutide emerged [228]. In the European EudraVigilance database (1 January 2018–10 July 2023) 41,236 individual case safety reports (ICSRs) for GLP-1 RA were retrieved, of which 230 (0.6%) described at least one suicidal event. The most frequently implicated drugs were liraglutide (38.3%) and semaglutide (36.5%), and the predominant event types were suicidal ideation (65.3%) and suicide attempts (19.5%). Disproportionality analysis showed significantly higher reporting odds for suicidal events with semaglutide versus dulaglutide (ROR 2.05; 95% CI 1.40–3.01) and versus exenatide (ROR 1.81; 95% CI 1.08–3.05), and an even stronger signal for liraglutide versus dulaglutide (ROR 3.98; 95% CI 2.73–5.82) and versus exenatide (ROR 3.52; 95% CI 2.10–5.92). Fatal outcomes were reported in 5.9% of these cases [229].

Therefore, pharmacovigilance studies have reported isolated cases of suicidal ideation, but predominantly among patients receiving concomitant psychotropic medications, suggesting that pre-existing psychiatric vulnerability may increase the risk of such adverse effects [230]. This uncertainty is reflected in regulatory inconsistencies: while U.S. prescribing information includes warnings to monitor for depression or suicidal thoughts when liraglutide is used for weight management [231], the equivalent European Summary of Product Characteristics lacks such statements [232].

The biological plausibility of a protective psychiatric effect stems from the multimodal actions discussed in Section 8. By suppressing pro-inflammatory cytokines (IL-1β, TNF-α) and enhancing neurogenesis via BDNF signaling, GLP-1RAs target the very drivers of depressive pathophysiology [8,33,124]. Rather than a risk factor, these agents may act as a protective buffer by mitigating the chronic low-grade inflammation and brain diabetes typical of metabolic disorders [12,33,34,174,175,233,234].

While causality cannot be established from spontaneous reports, these signals underscore the importance of monitoring mood, depressive symptoms, and suicidal thoughts, particularly during the first month of therapy.

## 10. Conclusions

GLP-1 and dual GLP-1/GIP agonists represent a pivotal advancement in the therapeutic landscape, marking a paradigm shift from purely metabolic management to multimodal neuropharmacological intervention. These agents redefine the intersection between systemic glucose regulation and central nervous system homeostasis.

In severe mental illness, incretin mimetics offer a unique clinical advantage. They mitigate the metabolic burden of antipsychotic therapy, specifically weight gain and insulin resistance, while simultaneously targeting the neuroinflammatory drivers of cognitive impairment. By restoring brain insulin signaling and inhibiting the pro-inflammatory AGE-Receptor for AGE axis, GLP-1 and GIP agonists demonstrate legitimate disease-modifying potential. Clinical evidence indicates that these therapies provide a protective buffer capable of slowing neurodegenerative progression, moving beyond mere symptomatic relief.

Future research must prioritize dedicated neurometabolic trials. Longitudinal neuroimaging is essential to confirm gray-matter preservation, while the modulation of reward circuits warrants exploration within addiction medicine. Finally, cost-effectiveness evaluations are necessary to ensure global access to these therapies at the frontier of integrated medicine.

## Figures and Tables

**Table 1:** GLP-1 R distribution: relative expression.

Species	Brain Region/Tissue	Predominant Cell Type (s)	Relative Expression	Sources
**Mouse**	Septal nucleus, hypothalamus, brainstem (including NTS)	Neuronal somata & projections	High	[64]
	Circumventricular organs, amygdala, ventrolateral medulla	Neurons (eYFP/tdRFP-positive)	High	[65]
	Cortex & hippocampus (areas without pre-proglucagon input)	Neurons (non-GFAP, some catecholaminergic)	Low–Moderate	[65]
**Rat**	Circumventricular organs, arcuate nucleus (ARC), nucleus tractus solitarii (NTS)	Neuronal perikarya, dendrites, axon varicosities	High	[66]
	Telencephalic & diencephalic nuclei, cerebellum	Neuronal profiles	Low–Moderate	[66]
	Peripheral-like axonal projections	Presynaptic axon terminals	Low–Moderate	[66]
**Human**	Frontal cortex (highest GLP-1R), hypothalamus, medulla oblongata, parietal cortex	Neuronal membranes	High	[67,68]
	Orbitofrontal cortex, cerebellum	Minimal/absent GLP-1R	Low	[68]
	Brainstem nuclei (e.g., dorsal vagal complex)	Neuronal cells	Moderate–High	[68]

## Data Availability

No new data were created or analyzed in this study. Data sharing is not applicable to this article.

## Abbreviations

The following abbreviations are used in this manuscript:

GLP-1	Glucagon-Like Peptide 1
GIP	glucose-dependent insulinotropic polypeptide
PC1/3	Prohormone Convertase 1/3
CNS	Central Nervous System
NTS	Nucleus of the Solitary Tract
GLP-1Rs	GLP-1 Receptors
VTA	Ventral Tegmental Area
NAc	Nucleus Accumbens
GABA	Gamma-Aminobutyric Acid
T2DM	Type 2 Diabetes Mellitus
GLP-1RAs	GLP-1 Receptors Agonists
GPCRs	G Protein-Coupled Receptors
PKA	Protein Kinase A
DPP-4	Dipeptidyl Peptidase-4
BBB	Blood–brain barrier
BDNF	Brain-Derived Neurotrophic Factor
GFAP	Glial Fibrillary Acidic Protein
GDNF	Glial-Cell-Line-Derived Neurotrophic Factor
IR	Insulin-Receptor
AGEs	Advanced Glycation End Products
ADDLs	Amyloid-Beta-Derived Diffusible Ligands
